# Alternative Woods in Enology: Characterization of Tannin and Low Molecular Weight Phenol Compounds with Respect to Traditional Oak Woods. A Review

**DOI:** 10.3390/molecules25061474

**Published:** 2020-03-24

**Authors:** Ana Martínez-Gil, Maria del Alamo-Sanza, Rosario Sánchez-Gómez, Ignacio Nevares

**Affiliations:** 1 Department of Analytical Chemistry, UVaMOX- Universidad de Valladolid, 34001 Palencia, Spain; anamaria.martinez@uva.es (A.M.-G.); rosario.sanchez@uva.es (R.S.-G.); 2 Department of Agricultural and Forestry Engineering, UVaMOX-, Universidad de Valladolid, 34001 Palencia, Spain

**Keywords:** alternative woods, ellagitannins, low molecular phenols, enology, traditional oak

## Abstract

Wood is one of the most highly valued materials in enology since the chemical composition and sensorial properties of wine change significantly when in contact with it. The need for wood in cooperage and the concern of enologists in their search for new materials to endow their wines with a special personality has generated interest in the use of other *Quercus* genus materials different from the traditional ones (*Q. petraea*, *Q. robur* and *Q. alba*) and even other wood genera. Thereby, species from same genera such as *Q. pyrenaica* Willd., *Q. faginea* Lam., *Q. humboldtti* Bonpl., *Q. oocarpa* Liebm., *Q. stellata* Wangenh, *Q. frainetto* Ten., *Q. lyrata* Walt., *Q. bicolor* Willd. and other genera such as *Castanea sativa* Mill. (chestnut), *Robinia pseudoacacia* L. (false acacia), *Prunus avium* L. and *P.* *cereaus* L. (cherry), *Fraxinus excelsior* L. (European ash) and *F. americana* L. (American ash) have been studied with the aim of discovering whether they could be a new reservoir of wood for cooperage. This review aims to summarize the characterization of tannin and low molecular weight phenol compositions of these alternative woods for enology in their different cooperage stages and compare them to traditional oak woods, as both are essential to proposing their use in cooperage for aging wine.

## 1. Introduction

Among oak woods, the most traditional *Quercus* genus species for wine aging are *Q. alba,* found on the USA east coast (the so-called American oak), and *Q. petraea* and *Q. robur* in the forests of France (the so-called French oak). The first two species are mainly employed to age wine while *Q. robur* is more common in alcoholic beverages such as cognac [1]. At present, there are two main market sources for oak barrels, the United States and France, though some other countries are increasing their production.

Customs in wine aging are changing since in many winemaking regions of the world the widespread use of new oak barrels (or those used for a limited period) is increasing. This trend is generating an increase in new barrel demand, which in turn has resulted in a need for exploring new sources of quality wood for cooperage [2]. Consequently, oaks from different European countries (Hungary, Poland, Russia, Ukraine, Slovenia, Romania, Moldova, Spain and Portugal) have entered the market as alternatives to traditional woods [2]. Most of them are of the same French species (*Q. petraea* or *Q. robur*) but sold at a lower price. Studies even state that these European oaks have characteristics half way between those of French and American oaks [3,4,5].

On the other hand, the use and/or study of alternative oaks (rather than the traditional ones) has been suggested as a solution to the search for new sources of quality wood for cooperage in order to conserve current areas and seek out woods which contribute distinct notes valued by the consumer. In this scenario, a market opportunity has opened up for oak species not traditionally used in cooperage such as *Q. faginea, Q. pyrenaica, Q. farnetto, Q. oocarpa* and *Q. humboldtii.* Moreover, the fact that the wine market is becoming more and more saturated and competitive means that enologists are interested in aging wines in barrels made of different woods in order to endow wines and wine-based products with a special personality. Therefore, the cooperage industry is obliged to offer the widest range of products. In the last few years, the enological use of different species of wood such as *Robinia pseudoacacia* L. (false acacia)*, Castanea sativa* Mill. (chestnut), *Prunus avium* L. and *Prunus cereasus* L. (cherry), *Fraxinus excelsior* L. and *F. americana* L. (European and American ash, respectively), among others, has been proposed as an alternative to oak [6,7,8,9]. Moreover, many producers even prefer using local woods in order to reduce costs [6] and recently some wine cellars have ordered barrels from cooperages with some non-oak staves included.

Oak chemical composition influences enological wine quality decisively. The oak cell wall components are cellulose, hemicellulose and lignin. These macromolecules, polysaccharides (cellulose and hemicellulose) and polyphenols (lignin) contribute physicochemical characteristics such as tensile strength, compressive strength and impermeability to this wood. The other components are called an “extractable fraction” and represent up to 10%-15% of dry wood. These compounds are difficult to classify because of their varied nature. Whereas ellagitannins are the most abundant components in oak, there are others with different chemical structures such as low molecular weight polyphenols (LMWP) and volatile compounds. Some of these compounds are the source of many of the interesting organoleptic characteristics found in aged wines and their presence encourages enologists to adopt this practice.

The aim of this review was to recapitulate and compare the composition in tannins and in low molecular weight phenol compounds of woods different from those traditionally used in enology (*Q. alba*, *Q. petraea* and *Q. robur*) in each of the different stages of cooperage (fresh wood, after seasoning and after toasting).

## 2. Wood Composition

Table 1 presents a summary of extraction and analysis methods of the compounds studied in the woods. We can observe that papers differ in the analyzed sample size, sample preparation, solvents used, wood/solvent ratio and extraction time.

### 2.1. Ellagitannins: Influence of Botanical Species on Their Concentration in the Woods Used in Cooperage

**Figure 1:**
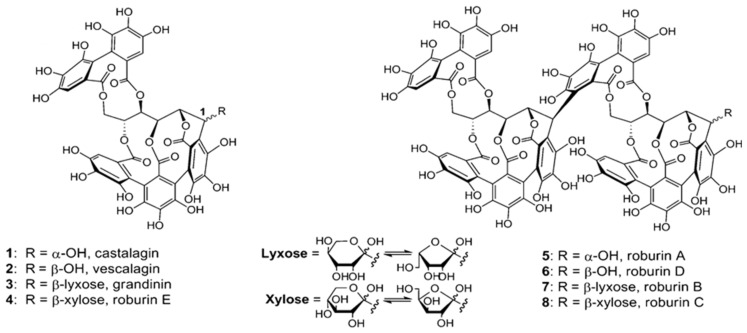
Structure of eight ellagitannins present in oak wood. Figure adapted from Jourdes et al. [49].

Ellagitannins may represent up to 10% of the heartwood. Eight ellagitannins have been identified in traditional oak species: castalagin, vescalagin, granidin and roburins (A, B, C, D and E) [16,21,33,34], whose structure is shown in Figure 1. Wood composition depends not only on species but also many other factors such as silvicultural, geographic origin and cooperage processing, which affect the extractable fraction [5,13,23,35]. Ellagitannins are transferred to the wine during aging, contributing to sensations of bitterness and astringency [36,37,38] and behaving as antioxidants due to their capacity to consume oxygen [39,40]. Moreover, ellagitannins directly affect wine color via reactions with anthocyanins forming red orange anthocyanin-ellagitannin complexes but are much more stable over time than free anthocyanins [41,42,43]. Ellagitannins also often occur in association with flavonoids to form flavono-ellagitannin derivatives (such as acutissimin A and acutissimin B) detected in aged wine [44,45] and are of interest due to their biological properties, such as their antitumor activity [46]. Ellagitannins are also involved in other chemical reactions, for example, in tannin condensation [47]. Moreover, these compounds are toxic to microorganisms, and prevent rapid decay of the wood, so an abundance in wood endows it with good resistance to fungal degradation [45,48].

The composition of ellagitannins in green wood, that is, when it has been cut without any additional treatment, indicates the characteristics peculiar to a species and whether it is suitable for aging wine, allowing the cooperage treatments to be adapted to each species. The wood used in cooperage usually goes through a natural drying stage in the open which means it is dehydrated, loses soluble substances such as ellagitannins, especially in the first few millimeters of each stave face and, to a lesser degree but uniformly, on the inner surface of the wood [16,33]. This decrease depends on the length of the drying period and phenomena like the lixiviation produced by rain or the water applied in cooperage and the oxidative hydrolytic degradation process [50], which involves the formation of free ellagic acid [16,51]. This hydrolysis is due to the significant enzymatic activity of a fungal nature [52] which occurs in wood and which significantly modifies its polyphenolic profile, releasing glucose through the destruction of heterosydic phenolic structures (coumarins and hydrolysable tannins) [53]. These biochemical reactions are affected by physical mechanisms associated with rainfall, UV radiation and variations in temperature (thermal amplitude) [50,54]. The elimination of these water-soluble phenolic compounds affects the decrease in the wood’s organoleptic characteristics of bitterness or astringency. It could even be related to the concentration of extractable water-soluble substances, as those are capable of occupying certain cell wall pores, which were full of water prior to drying, due to wood contraction [55]. Toasting is the final process to which the wood is subjected before entering into contact with the wine. During this treatment the ellagitannins decrease, thus increasing the ellagic acid concentration: this is dependent on the toasting conditions [16,35,56].

The ellagitannin concentration in different alternatives to oak are shown in Table 1, Table 2 and Table 3 in green, after drying and after toasting, respectively. Moreover, the ellagitannins in traditional woods have also been included when comparisons have been made.

#### 2.1.1. Alternative Wood Species from *Quercus genus*

Within the *Quercus* genera, *Q. pyrenaica* has been studied the most in the last few years and research on detailed ellagitannin concentration in *Q. faginea, Q. humboldtii, Q. farnetto, Q. stellata* and *Q. oocarpa* has been found. The main phenolic components analyzed in the green and seasoned wood of these *Quercus* were ellagitannins, with similar results to those found in other oaks traditionally used in enology [11,12,13,19,21,24,25,33].

*Q. pyrenaica, Q. faginea* and *Q. humboldtii* green wood present the eight ellagitannins identified in the oaks normally used enologically. Monomers are more abundant than dimers in the three species as occurs with traditional oaks. The % of castalagin and vescalagin in all ellagitannins is similar in all the species (alternative and traditional oaks). The ellagitannin composition of *Q. pyrenaica* is very similar among those of different Spanish origins studied. *Q. pyrenaica* and *Q. faginea* total ellagitannin concentration is between those of *Q. robur* and *Q. alba* and like *Q. petraea* (Table 2).

However, the concentration in *Q. humboldtii* is less than that of other species and more closely resembles that of *Q. alba*.

Among the *Quercus* woods studied after drying, *Q. frainetto* is distinguished by its greater concentration of pentosylated dimers [24], as roburin B and C are the predominant ellagitannins of this species (Table 3). In addition, *Q. frainetto* is high in roburin A concentration in comparison with both the traditional and other species, and roburin D concentrations are similar to those of *Q. pyrenaica* but higher than those of other species studied (other alternative species and traditional oaks). Similarly, this species from Hungary contains concentrations of castalagin, vescalagin, roburin E and granidin similar to those of traditional species and *Q. petraea*, although slightly higher, which means it has the highest ellagitannin concentration (108 mg/g) of those studied. At the other extreme is *Q. humboldtii*, the species with the lowest total ellagitannin concentration (1.61 mg/g). Bearing in mind intra-species variability, its ellagitannin composition is similar to that of *Q. alba* [13].

As regards *Q. pyrenaica,* all the authors describe this wood´s concentration after drying (regardless of treatment time and method) as being between that of *Q. robur* and *Q. petraea*, the same as that observed in green wood and this occurs in the case of *Q. faginea* oak after 12 and 36 months´ drying (Table 3). Castalagin and vescalagin are the main ellagitannins in these two species after drying except for *Q. pyrenaica* from Álava (Spain), in which the roburin E and granidin concentrations are higher than those of vescalagin after drying, regardless of length (12 or 36 months) and of *Q. faginea* from Álava (Spain), though only after 36 months´ drying. The work done by Alañon et al. [21] records very low ellagitannin concentrations in the oven-dried wood (drying in oven to 0% internal humidity) of *Q. pyrenaica* (2.81 mg/g) as well as *Q. petraea, Q. robur* and *Q. alba* (1.98, 3.93 and 0.88 mg/g, respectively). The ellagitannin concentration of the *Q. pyrenaica* from Álava (Spain) is between 19.75 mg/g after 12 months´ drying and 16.1 mg/g after 36 months (Table 3). The variability found in the forests of Gerês, Portugal, is greater as some authors record 77.9 mg/g and others 17.87 mg/g after the same drying period of 24 months in the open air [23,25], which indicates great intra-species variability as observed in traditional species. Finally, this species from the forests of Guarda, Portugal, presents concentrations of 54.28 mg/g (Table 3). In the case of *Q. faginea* only one origin (Álava, Spain) has been studied recording very similar concentrations regardless of drying: 26.97 and 24.11 mg/g after drying in the open for 12 and 36 months, respectively.

Concentrations similar to that of *Q. petraea* (66.9 mg/g) are found in *Q. stellata*, although roburin D is not detected. The main ellagitannins in this species are the monomers, vescalagin and castalagin with 69% of the total. In contrast the total concentration in *Q. oocarpa* is 39.3 mg/g, similar to that found in *Q. petraea* and *Q. alba*, consisting of monomer ellagitannins (both pentosylates and non-pentosylates), similar to those found in *Q. alba* by the same author [24]. Therefore, all these species except *Q. frainetto* follow the same pattern as traditional woods with the main ellagitannin being first castalagin, then vescalagin, granidin and roburin C. Another species studied is *Q. cerris*, in which no ellagitannins and only traces of ellagic acid can be detected [57]. This species mainly has condensed tannins, which account for up to 3% of the wood´s dry weight. The insoluble fraction in this species represents over 95% in heartwood [57].

As regards concentration in toasted wood, results have only been found for *Q. pyrenaica, Q. faginea* and *Q. humboldtii*. Coinciding with the above, toasted *Q. pyrenaica* and *Q. faginea* woods have an ellagitannin profile similar to that of traditional European species, especially *Q. petraea*, and higher than that of *Q. alba*, while *Q. humboldtii* presents lower concentrations similar to those of *Q. alba* [13]. The range of total ellagitannin concentrations found in *Q. pyrenaica* is very wide (4.32 to 47.05 mg/g, Table 4); wood from the forest of Gerês differs the most with the same drying and very similar toasting, since they only differ in time (10 min longer), thus indicating great intra-species variability. *Q. faginea* and *Q. humboldtii* are reported to present 9.34 mg/g and 0.12 mg/g, respectively, after toasting. On comparing the two species studied from the forests of Álava (Spain), their total ellagitannin concentrations are very similar: 6.37 and 9.34 mg/g for *Q. pyrenaica* and *Q. faginea,* respectively. The *Q. faginea* wood from Álava presents higher castalagin, vescalagin, roburin E and D concentrations than that of *Q. pyrenaica.*

In general, the significance of each ellagitannin in the *Quercus* species studied has the same profile after toasting and on drying, with castalagin being quantitatively of greater importance in comparison with the others, followed by the other monomers such as vescalagin and other pentosylates (granidin and roburin E).

The variation in ellagitannin concentration in the same wood due to the different cooperage processes (green, drying or toasting) has been reported in various papers. Fernández de Simón et al. [33] studied *Q. pyrenaica* and *Q. faginea* from Álava (Spain) green wood and after drying. The ellagitannin concentration of these species after 12 months´ drying in open air decreases from 28.12 to 19.75 mg/g in *Q. pyrenaica* and from 32.51 to 26.97 mg/g in *Q. faginea* (Table 1 and Table 2) as do the traditional species. The ellagitannins in *Q. pyrenaica* that degrade most are vescalagin and roburins A, B and C, with losses of 57, 47, 47 and 59% in comparison with their initial concentration, while roburins A, C and D decrease in *Q. faginea* by 79, 66 and 60%. Jordao et al. [23] reported that toasting affects the ellagitannin concentration in the *Q. pyrenaica* of both origins, decreasing 41% and 13% in the forests of Gerês and Guarda (Portugal), respectively, after toasting at 160–170 °C for 20 min and 82 and 42% after a more intense treatment at 250–260 °C for 27 min. Therefore, the higher the toasting intensity, the more degradation as occurs in traditional species. In the wood from the forest of Gerês (Portugal) vescalagin, roburin E and castalagin degrade more after lower intensity toasting with losses of 51, 44, 44%, respectively, in comparison with initial concentrations. However, more intense toasting degrades roburins E and D completely while granidin and vescalagin losses are 84% and that of castalagin is 71%. As regards wood from the forest of Guarda (Portugal) roburin D degrades completely after both treatments, followed by granidin with a 51% loss of its initial concentration after light toasting (160–170 °C for 20 min) and 76% after more intense toasting (250–260 °C for 27 min). Intense toasting in woods from this forest degrades all the ellagitannins as occurs with those of the wood from Gerês; however, light toasting of the Guarda wood does not decrease the concentration of castalagin, vescalagin and roburin E (Table 3 and Table 4). Castro-Vázquez et al. [25] observed that the *Q. pyrenaica* wood from the forest of Gerês (Portugal) also decreases 13.55 mg/g after toasting at 160–170 °C for 30 min, with both the pentosylated and non-pentosylated monomers degrading the most: over 71% of their initial concentration.

Some authors speak of the castalagin/vescalagin ratio as being characteristic of the species [21,24]. However, as shown in Table 1, Table 2 and Table 3 this cannot be guaranteed as there is a wide variety of results for this ratio within the same species. In the same way, the different cooperage treatments are not clearly significant for this ratio. The range of ellagitannin concentrations within the same species of wood is very wide so the significance of wood treatment and intra-species variability can be observed.

#### 2.1.2. Alternative Wood Species from Genus Different to *Quercus*

*Robinia pseudoacacia* (acacia) [6,17] and *Fraxinus americana* or *Fraxinus excelsior* (ash) [6,18] heartwoods do not present any hydrolysable tannins in their composition. Alañon et al. [21] found that *Prunus avium* (cherry) presents very low concentrations of tannins after drying in an oven with castalagin and vescalagin concentrations of 0.04 mg/g and 4.19 µg/g, respectively. However, Sanz et al., 2011 [17] did not find ellagitannins in cherry wood composition after natural drying for 24 months or after toasting at 2 intensities (165 °C for 35 min or 185 °C for 45 min). This is a significant qualitative difference when compared with the composition of traditional oak, since these species would not provide the hydrolysable tannins that the traditional oaks would and therefore the chemical reactions which the ellagitannins usually participate in during aging would not occur. However, cherry and acacia have condensed tannins in their composition (these tannins have also not been detected in ash), which are not detected in oak and different to those normally found in wine (to be discussed in Section 2.3).

Among the species studied other than *Quercus,* chestnut is the only one containing ellagitannins (Table 3 and Table 4). Five studies describe the composition of *Castanea sativa* Mill. (chestnut) in dry wood in detail [15,21,22,24,25] (Table 3) and two that in toasted wood (3 toasting degrees) [15,25] (Table 4). However, no work on green wood has been found. Chestnut after seasoning, either in the oven or naturally, but not for a specific time, presents the 8 ellagitannins. After 24 months´ drying the 8 ellagitannins are only quantified in the chestnut from Portugal [25]; however, some of them are either not detected or not studied in the wood from France [15,24] (Table 3). In addition, the total concentration of ellagitannins in chestnut wood seasoned in the two forms indicated above is higher than in traditional oaks (Table 3). Nevertheless, after 24 months drying the total ellagitannin concentration is similar to that found in traditional oaks, especially *Q. petraea* (Table 3). Vescalagin and castalagin are the most important ellagitannins in chestnut wood, representing 75–100% of total ellagitannins (Table 3 and Table 4), as is the case in traditional oak. Castalagin is the main ellagitannin monomer found in traditional oak samples and Sanz el al. [15] observed the same for chestnut. However, Alañon et al. [21], Viriot et al. [22] and Vivas and Glories [24] found that vescalagin is the main ellagitannin in this species. Castro-Vázquez et al. [25] reported the same result in dry wood, although after toasting the concentrations of both ellagitannins are very similar (Table 2 and Table 3) with a loss of vescalagin from 20.3 mg/g to 4.32 mg/g. As can be observed, and as occurs in oak [13,23,59], toasting chestnut decreases the concentration of ellagitannins and this effect is accentuated on increasing the toasting level. The total ellagitannin concentration is reduced from 43.73 mg/g in seasoned wood to 10.15 mg/g after light toasting and to 0.66 mg/g after more intense treatment [15] (Table 2 and Table 3) or, according to Castro-Vázquez et al. [25] from 31 mg/g to 10.51 mg/g after toasting (Table 2 and Table 3). Acutissimin A is identified in the chestnut heartwood by Sanz et al. [15] in both dry (3.3 mg/g) and toasted wood after light (3.9 mg/g) or medium toasting (0.2 mg/g).

### 2.2. Low Molecular Weight Phenol (LMWP): Influence of Botanical Species on Their Concentration in the Woods Used in Cooperage

As previously commented, green wood cannot be used for cooperage since it contains high moisture and its extractable compounds are not compatible with the objective of improving the quality of wine. In contrast to what happens with ellagitannins, low molecular weight compounds (LMWP) increase their concentration in traditional woods during natural drying in the open and after toasting [33,50]. The main phenolic acid compounds identified in seasoned and toasted oaks (*Q. petraea, Q. robur* and *Q. alba*), are acids, specially hydroxybenzoic [3,7,13,28].

**Figure 2:**
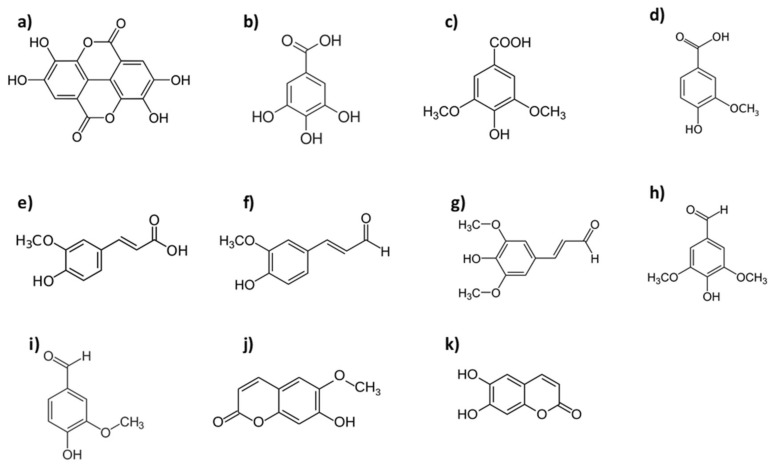
The main low molecular weight phenol (LMWP) identified in oaks: acids: (**a**) ellagic, (**b**) gallic, (**c**) syringic, (**d**) vanillic, (**e**) ferulic; aldehydes: (**f**) coniferyl, (**g**) sinapic, (**h**) syringic, (**i**) vanillin; coumarins: (**j**) scopoletin, (**k**) aesculetin. (https://en.wikipedia.org/wiki/).

LMWP (acids, aldehydes and coumarins) analyzed by high performance liquid chromatography (HPLC) are shown in Table 4 and their molecular structures can be seen in Figure 2, where the results are related to green wood; Table 5 summarizes the seasoned wood results; and Table 6 those for toasted *Quercus* species (*Q. pyrenaica, Q. faginea* and *Q. humboldtti*) and other genera (*Castanea sativa, Robinia pseudoacacia, Prunus, Fraxinus, Alnus* and *Fagus*). In these Tables, data on traditional oaks appear when comparisons have been made with alternative woods in the studies mentioned.

#### 2.2.1. Alternative Wood Species from *Quercus* Genus

In green wood, LMWP have been studied in *Q. pyrenaica, Q. faginea* and *Q. humboldtii*. Seven different habitats of *Q. pyrenaica* have been studied from two Spanish regions: six from Castile and León (Gata/Peña de Francia, Guadarrama, Cantabrian mountains, Iberian mountain range, Alitse-Maragatería and Gredos/Ávila mountains) [11,14]; and one from the Basque country (Álava) [20,33]. In all of these species, the main component is ellagic followed by gallic acid (Table 5), as usually happens in traditional oaks. In general, the acid concentration in the three species is higher than that of aldehydes, following the same pattern as in traditional species. However, vanillin and syringaldehyde concentrations are higher than their corresponding acids except for vanillin in *Q. pyrenaica* from the Iberian mountain range [11] and *Q. faginea* from Álava [20,33]. Total aldehyde concentration in the alternative woods is as in traditional ones, the two species from Álava being the ones with the lowest concentrations, especially in aldehydes with the most influence on the sensory characteristics of the wines: syringaldehyde and vanillin. However, the total acid concentration in *Q. pyrenaica* is higher than in traditional woods, except for those from Gredos and Álava (Table 5). Green wood of *Q. pyrenaica* from Castile and León has a higher ellagic acid concentration than traditional woods (Table 5). However, the same species from Álava presents concentrations similar to the woods normally used in enology. Moreover, this wood from Álava has the lowest concentrations of syringic acid, syringaldehyde, sinapaldehyde and vanillin in comparison with the woods from Castile and León. Therefore, the *Q. pyrenaica* species wood from Castile and León is richer in LMWP, concentrations of 1061 to 697 µg/g, than that of Álava with 262 µg/g. Moreover, the LMWP concentration of the *Q. pyrenaica* wood from Castile and León is also higher than that of the traditional ones, except for that from Gredos (Ávila). The *Q. faginea* green wood from Álava presents LMWP quantities similar to *Q. pyrenaica* from Álava (Table 5) and lower quantities of syringic, vanillic and ferulic acids and of sinapaldehyde and vanillin, but higher ones of gallic acid than in traditional oaks. The green wood of *Q. humboldtii* shows concentrations of aldehydes and acids similar to those of *Q. petraea*, *Q. robur* and *Q. alba*.

Fernández de Simón et al. [20,33] described another two types of low molecular weight compounds, called A and B, in some of the alternative species (*Q. pyrenaica* and *Q. faginea*) and two of the traditional ones (*Q. petraea* and *Q. robur*). The A compounds, whose UV spectra are similar to those of ellagic acid, are present in all the species and in similar quantities. However, some of the B compounds, whose UV spectra are similar to those of gallic acid, are found in traditional woods but not in all the alternative species: B2, B3 and B4 are not present in *Q. pyrenaica* nor are B1 and B2 found in *Q. faginea*. The B compounds found in all the species are generally more abundant in the alternatives (*Q. pyrenaica* and *Q. faginea*) than in the traditional oaks (*Q. petraea* and *Q. robur*). These authors suggest that the forests of these two new species could be distinguished via the ratio of these two types of compound. In addition to green wood Fernández de Simón et al. [33] studied wood dried for 1 year in the open, observing that the behavior of compounds A and B after drying is not clear as they increase in some cases, even detecting compounds not present in the green wood after drying (like B14, 15, 16, 17), and in other cases decrease to the point of non-detection. Compounds B2 and B4 are not detected in dry *Q. pyrenaica* but all the other A and B compounds increase their concentration after this treatment except B9 which decreases to non-detectable concentrations [33]. In addition, the seasoned *Q. pyrenaica* wood presents higher quantities of all the compounds in comparison with *Q. robur*, *Q. petreae* and *Q. faginea*, except for A3 in *Q. petraea and Q. robur* and B16 in *Q. faginea*. The dry wood of this species presents compound B1, which is not detected in the dry wood of any other species. B6, B7, B13, B14 and B17 are only detected in the dry wood of the new species (*Q. pyrenaica* and *Q. faginea*) but not in the traditional ones [33]. Drying decreases 13 of the 27 A and B compounds analyzed in *Q. robur,* with 10 of them not being detected. In *Q. petraea* this treatment decreases 11 of them with 6 not being detected; and the concentration of 7 decreases in *Q. faginea* with 4 being undetectable [33].

Table 6 shows that the acid concentration in seasoned *Q. pyrenaica*, *Q. faginea* and *Q. humboldtii* woods is still higher than the aldehydes with ellagic and gallic acids being the principal ones. Acid and aldehyde concentration in *Q. pyrenaica* and *Q. faginea* from Álava is higher with a longer seasoning time (Table 6). As the data are not taken from the same study it cannot be predicted with any certainty that longer drying means a higher concentration, but everything seems to indicate that it is probably a decisive factor. Ellagic acid concentration in *Q. pyrenaica* is 137 µg/g when oven dried; 1254.8 to 2679 µg/g when dried in the open without specifying time (although it is probably a long period given the rather high concentrations); and 299, 297 and 735 µg/g after 12, 24 and 36 months´ drying in open air, respectively; time again seems to be a very significant factor in LMWP concentration. On comparing *Q. pyrenaica* and *Q. faginea* from Álava after 12 and 36 months drying, *Q. pyrenaica* wood presents higher concentrations of all the LMWP except ellagic and ferulic acids, sinapaldehyde after 12 months and ellagic, gallic and ferulic acids after 36 months. As regards *Q. humboldtii* wood, Martínez-Gil et al. [60] observe that LMWP concentration is similar to that found in *Q. petraea* and *Q. alba,* though with higher concentrations of gallic acid and sinapaldehyde, and lower ones of syringic acid and coniferaldehyde.

Fernández de Simón et al. [33] studied the effect of drying on the composition of *Q. pyrenaica* and *Q. faginea* woods showing that LMWP concentrations increased considerably (Table 4 and Table 5). This study observed that gallic and ellagic acid content more than double their concentration due to drying in the open for one year, and even multiply gallic acid concentration by 7 in *Q. pyrenaica* (Table 4 and Table 5). The only LMWP which decreases on drying is coniferaldehyde in *Q. faginea* wood. However, the concentration of five LMWP: gallic and ferulic acids, sinapaldehyde, scopoletin and aesculetin, decreases in *Q. petraea* wood during the same type of drying [33].

Vivas et al. [24] studied the composition of different *Quercus* (*Q. frainetto, Q. stellata* and *Q. oocarpa)* woods after drying in open air for 24 months and stated that the ellagic acid concentration is greater than that of gallic acid in *Q. stellata* and *Q. oocarpa,* as usually occurs in traditional oaks, but is the opposite in *Q. frainetto* [24]. These authors indicated higher ellagic acid concentrations (11.2 mg/g) in *Q. stellata* than in the other two species (4.2 and 0.6 mg/g in *Q. oocarpa* and *Q. frainetto,* respectively), but lower than *Q. alba* (18.4 mg/g) and much higher than traditional European oaks (1.9 and 3.1 mg/g in *Q. robur* and *Q. petraea,* respectively).

*Q. frainetto* wood shows the highest concentration (3.2 mg/g) of gallic acid, more than double that of traditional oaks (1.4, 1.1 and 1.2 mg/g in *Q. robur, Q. petraea* and *Q*. *alba,* respectively) and also higher in *Q. stellata*, but with a smaller difference (1.8 mg/g). However, concentration in *Q. oocarpa* is similar to that of traditional woods with 1.3 mg/g.

LMWP concentration of the woods after toasting has only been studied in detail in *Q. pyrenaica*, *Q. faginea* and *Q. humboldtii* (Table 7). It has been stated that acid concentration remains higher than that of aldehydes in *Q. pyrenaica* and *Q. faginea* with the main ones being ellagic and gallic acids. Nevertheless, the opposite occurs in toasted *Q. humboldtii* wood, as the aldehyde concentration is greater, the main one being sinapaldehyde (Table 7). This behavior has also been observed in traditional woods, especially *Q. alba* and/or *Q*. *robur* [3,13,30,32] (Table 7), although the most common profile is that observed in *Q. pyrenaica* and *Q. faginea.* The total LMWP concentration interval in *Q. pyrenaica* wood is from 607 to 20500 µg/g, while total LMWP concentrations of 2132 µg/g and 2464 µg/g, respectively, are found in *Q. faginea* and *Q. humboldtii* (Table 7). As regards how toasting affects the LMWP of these alternative species, the studies of interest are those which show results before and after this treatment [3,13,25,27], one even showing results after two toasting intensities (100 °C or 150 °C for 45 min) [27]. Gallic acid concentration decreases in the woods of all the species (Table 5 and Table 6): between 6 and 25% in *Q. pyrenaica*, 25% in *Q. faginea* and 68% in *Q. humboldtii*. Moreover, gallic acid degradation in *Q. pyrenaica* wood is greater with a higher toasting temperature (Table 7). However, ellagic acid increases in all these except *Q. humboldtii*, which maintains practically the same concentration (Table 5 and Table 6). Vanillic and syringic acid concentrations in woods also increase in the three species during toasting, except in the Castro-Vázquez [25] study, where these two acids decrease in *Q. pyrenaica* wood. Ferulic acid has only been studied in *Q. pyrenaica* [25,27], and a significant decrease (15 to 44% of its initial concentration) is observed in both articles, this being greater as the temperature rises [27]. Aesculetin concentration diminishes in *Q. pyrenaica* and increases in *Q. faginea* (Table 5 and Table 6). However, the compounds really affected by toasting are the aldehydes, as very significant increases are observed: *Q. humboldtii* goes from 18 to 1778 µg/g, *Q. faginea* from 26 to 670 µg/g and *Q. pyrenaica* from 73 to 544 µg/g according to Canas et al. [27], from 46 to 909 µg/g according to Cadahía et al. [3] and from 43 to 91 µg/g according to Castro-Vázquez et al. [25]. The same occurs with the traditional species since acid concentration increases slightly during toasting, but the aldehydes present a really considerable increase [3,13]. 

#### 2.2.2. Alternative Wood Species from Different Genus to *Quercus*

*Castanea sativa* Mill.:

Seasoned chestnut wood is the richest in LMWP of the non-*Quercus* species, both in acids and in aldehydes (Table 6). The acid concentration is higher than that of aldehydes, as observed in *Quercus* woods. Ellagic and gallic acids are the most abundant compounds within this group, the gallic acid concentration generally being higher than that ellagic acid, in contrast to what occurs in *Quercus* species. The lowest gallic acid concentrations in dry wood have been found by Alañon et al. [21] in woods from Lugo (Spain), dried in an oven, and by Castro-Vazquez et al. [25] in woods from Gerês forest (Portugal) dried in open air for 24 months, with concentrations of 1155 and 1172 µg/g, respectively. Alañon et al. [21] found significant protocatechuic acid, 4-hydroxybenzoic acid and coumaric acid concentrations in dry wood, and even quantities higher than that of ellagic acid. However, other authors who also analyzed protocatechuic acid in this wood record much lower concentrations than the 113 µg/g found by Alañon et al. [21] and the 5 µg/g quantified by Sanz et al. [15]. Canas et al. [28,29] did not observe any furanic derivatives in seasoned chestnut wood or in the traditional woods. Nevertheless, Soares et al. [31] found 5-methylfurfural in seasoned chestnut wood and 5-methylfurfural and furfural in toasted wood, but at lower concentrations than oak. These authors [31] did not find 5-hydroxymethylfurfural but the toasted chestnut woods studied by Canas et al. [30] present 5-methylfurfural, furfural and 5-hydroxymethylfurfural, even at concentrations higher than oak. The highest total LMWP concentrations in dry wood are 14730 µg/g (18 months natural seasoning) [26] and in toasted wood 35282 µg/g (185 °C for 60 min) [32]. 

Toasting considerably decreases the concentration of gallic acid in chestnut wood. Sanz et al. [15] observed an increase of this compound in wood after toasting at 160-170 °C for 35 min, though the wood after toasting at 185 °C for 45 min presents a diminished concentration, also observed by the rest of the authors. However, the ellagic acid concentration in the wood increases with toasting [15,25,31,32], and is greater after more toasting [15,31]. In addition, syringic and vanillic acids increase during toasting, although Castro-Vázquez et al. [25] observed the opposite. The aldehydes are the compounds with the greatest increase during toasting, as also occurred in the *Quercus* species. It is also observed that the most important aldehyde from an organoleptic viewpoint, vanillin, due to the vanilla notes it contributes, increases considerably on toasting chestnut wood. In the studies where chestnut is compared to *Quercus* woods, it has been shown that vanillin concentration in chestnut is higher than in *Quercus* oak (Table 7). Similarly, it has been found in the literature that when the same liquid (wine, vinegar or brandy) is aged in chestnut and oak barrels the levels of vanillin detected in those from chestnut are higher than in those aged in traditional oak [61,62,63].

*Robinia pseudoacacia* L.:

The total LMWP percentage in comparison with the rest of the components found in acacia wood is low since the main compounds of this wood are flavonoids followed by condensed tannins [17] (described in Section 2.3). The two main compounds in dry acacia wood are hydroxycinnamic derivative compounds [17], with concentrations of 2506 and 437 µg/g. Magel et al. [64] also observed a hydroxycinnamic derivative as the main LMWP. These hydroxycinnamic derivatives decrease after light toasting (165° for 20 min), although they continue to be the main LMWP quantitatively. However, after medium toasting (165 ° for 30 min) there is only one of them and after medium plus toasting (185° for 45 min) both are completely degraded [17]. The following most important component quantitatively in dry acacia wood is gallic aldehyde with 108 µg/g, which increases on light and medium toasting to 137 and 245 µg/g, respectively, but is degraded on more intense toasting: 21 µg/g in medium plus toasting [17]. Significant concentrations of β-resorcilyc aldehyde are also found in dry wood (48 µg/g), increasing as a result of the toasting process [17]. So, after medium toasting (165 °C for 35 min), significant concentrations of coniferaldehyde and sinapaldehyde (276 and 239 µg/g, respectively) are maintained [17]. They increase with stronger toasting (165 °C for 35 min), especially the sinapaldehyde, going from 300 to 1666 µg/g (Table 7). This was also described by Soares et al. [31] with more intense toasting (240 °C for 120 min) (Table 7). However, Jordao et al. [7] did not detect coniferaldehyde in this wood after medium toasting. Another major compound found after a medium plus toasting treatment (185° for 45 min) is syringaldehyde, with concentrations of 326 µg/g [17], though this compound is not detected in dry or toasted wood in the other studies (Table 7). Vanillic acid has not been detected in any of the works in which dry or toasted acacia wood composition is analyzed by HPLC (Table 5 and Table 6) [6,7,17,31]; however, it is a characteristic acid in traditional oak. Protocatechuic aldehyde is found in both dry and toasted acacia wood [7,17,31]. Yet there is no clear behavior of this compound with the degree of toasting applied to the wood, since Soares et al. [31] observed a slight decrease with an increased toasting level while Sanz et al. [17] observed an increase with medium toasting and a decrease with light and medium plus toasting. As regards the furfurals, Soares et al. [31] reported very low concentrations of furfural and of 5-hydroxymethylfurfural in acacia wood in comparison with oak. Moreover no 5-methylfurfural is found in dry or toasted wood. Nor is *p*-coumaric acid detected in toasted acacia wood [7].

*Prunus avium* L. and *Prunus cereaus* L.:

The LMWP in cherry wood have been studied in two subspecies, *P. avium* and *P. cereaus*. The ellagic acid in the dry wood of *P. avium* is detected at very low concentrations (15.80 µg/g) in comparison with traditional oak [21] or even not detected [8]. The dry wood of *P. cereaus* presents a higher concentration of ellagic acid (194 µg/g) than those of *P. avium*, but lower than those found in naturally dried traditional wood (Table 6). This also decreases with toasting, as Soares et al. [31] observed that concentration in *P. cereaus* goes from 194 µg/g to 89 µg/g after toasting at 160 °C for 20 min, degrading completely when thermal treatment increases (200 °C for 120 min or 240 °C for 120 min) (Table 5 and Table 6). Ellagic acid is only detected in toasted *P. avium* wood [7] and, moreover, at higher concentrations than those found in dry wood (Table 5 and Table 6), so this subspecies of the central zone of France is probably richer in this compound. Gallic acid is only detected in the dry wood of the subspecies *P. avium* and at insignificant concentrations in comparison with oak (31.11 and 1.22 µg/g Table 6). The major LMWP in dry *P. avium* wood, according to Sanz et al. [8] are methyl syringate and 3,4,5-trimethoxyphenol, followed by *p*-coumaric acid and protocatechuic acid. According to Alañon et al. [21] they are: coniferaldehyde with 332.59 µg/g and sinapic acid with 106.8 µg/g, followed by sinapaldehyde with 78.72 µg/g and syringaldehyde with 42.01 µg/g as opposed to 36.06 µg/g of protocatechuic acid or 7.11 µg/g of coumaric acid. 

On the other hand, the main compound in toasted cherry wood is sinapaldehyde, with concentrations of 553 up to 1637 µg/g in *P. avium* and from 156 to 619 µg/g in *P. cereaus* (Table 7). Syringaldehyde and coniferaldehyde are the following compounds in abundance in toasted *P. avium* wood [8,32]; however, these were not detected by Jordao et al. [7] who describes *p*-coumaric acid as the main LMWP. The next major component in toasted *P. cereaus* wood is protocatechuic aldehyde, and in those woods subjected to high level toasting it is also syringaldehyde [31]. Cherry wood also presents appreciable quantities of benzoic acid [8], which may explain the high ethyl benzoate concentration found in the vinegars obtained by acidification in cherry wood barrels in comparison with those found when using other woods [61]. The quantities of vanillin found in toasted *P. avium* wood vary from 41.9 to 313.33 µg/g and in *P. cereaus* from 56.67 to 117.71 µg/g, in general being higher than those found in acacia, and somewhat lower than those recorded in ash, chestnut and oak (Table 7).

*Fraxinus americana* L. and *Fraxinus excelsior* L.:

Gallic acid and ellagic acid are not found in ash, the major compound in the dry wood being tyrosol, with 139 and 100 µg/g in *F. americana* and *F. excelsior,* respectively. However, this compound has not been previously detected in oak [18]. Tyrosol decreases during toasting, while coniferaldehyde and sinapaldehyde increase and are the major compounds in toasted *F. Americana* wood followed by vanillin and syringaldehyde [18]. This was also described for *F. excelsior* by Rodríguez Madrera et al. [32], while Sanz et al. [18] indicated that sinapaldehyde and syringaldehyde, followed by coniferaldehyde and vanillin, are the main compounds. The dry wood of *F. excelsior* has been described as being poorer in all the LMWP than *F. americana* (Table 6), although *F. excelsior* is richer in syringaldehyde, sinapaldehyde, vanillin, syringic acid and ferulic acid than *F. americana* after the same toasting, and *F. americana* presents greater concentrations of coniferaldehyde and vanillic acid (Table 7). Vanillin concentrations in toasted wood varies between 245 and 329 µg/g in *F. americana* and 222 and 404 µg/g in *F. excelsior*, concentrations generally similar or even higher than those found in traditional oaks (Table 7). Vanillic acid and protocatechuic acid are not detected in *F. excelsior* from Galicia, Spain [32], but are by Sanz et al. [18] although the origin of this wood is not stated. Finally, it should be mentioned that hydrotyrosol is detected in dry *F. excelsior* wood but not in any other (oak, cherry, acacia or chestnut) [6,18].

*Alnus glutinosa* L. and *Fagus sylvatica* L.:

Detailed LMWP composition in these two species was only found in one study [32], which looked at the wood with two toasting intensities. The major compounds described are coniferaldehyde and sinapaldehyde in both species and after both toastings, although protocatechuic acid is also quantified in *F. sylvatica*. These authors did not find protocatechuic acid in any of the oaks studied [32], nor did they find 4-hydroxybenzaldehyde, which is quantified in *A. glutinosa* and *F. sylvatica*. Coniferaldehyde, sinapaldehyde, syringaldehyde and vanillin concentrations are higher in oaks than in *A. glutinosa* and *F. sylvatica* (Table 7).

### 2.3. Other Compounds: Influence of Botanical Species on Their Concentration in the Woods Used in Cooperage

The most abundant phenolic compounds in traditional oak wood as well as in the new *Quercus* (*Q. faginea, Q. pyrenaica, Q. farnetto, Q. oocarpa* and *Q. humboldtii*) are ellagitannins, low molecular weight phenols and volatile phenols. However, oak heartwood does not contain other kinds of phenolic compounds, for example, flavonoids or condensed tannins [8,65]. This section will briefly discuss the compounds found in the new species (*Castanea, Robinia, Prunus and Fraxinus*) which have not been recorded in traditional oaks.

#### 2.3.1. *Castanea sativa* Mill.

This wood is characterized by being rich in hydrolysable tannins and poor in condensed tannins like the traditional woods. However, not only ellagitannins are found within the hydrolysable tannins, as in oak wood. Moreover, other hydrolysable tannins are present in this wood: galloyl and ellagic derivatives; specifically, 28 tannins, of which 23 are gallotannins and 5 ellagic derivatives. Most have been quantified in dry wood (34 months in open air), but not in toasted wood since this process causes degradation of these tannins. Gallotannins suffer greater degradation with the degree of toasting, going from 1908 μg/g in dry wood to 4047 μg/g in lightly toasted wood (165 °C for 35 min) and to 238 μg/g after medium toasting (185 °C for 45 min) [15]. Most ellagic derivatives also degrade during toasting and more so with higher level toasting; however, two ellagic derivatives increase with the degree of toasting, in such a way that total ellagic derivatives go from 411 μg/g in dry wood to 260 μg/g after light toasting and 263 μg/g after medium toasting [15].

A total of 27 compounds not found in oak are recorded in dry wood: 23 are gallotannins and 4 ellagic derivatives. The concentrations of gallotannins and ellagic derivatives in dry wood vary from 26 to 3270 μg/g and 37.4 to 250 μg/g, respectively [15]. The gallotannins found are methyl gallate (144 μg/g), digalloyl glucose (139 μg/g), 4 digalloyl-HHDP-glucose (880 μg/g), 5 trigalloyl glucose (3844 μg/g), trigalloyl-HHDP-glucose (98 μg/g), 7 tetragalloyl glucose (4636 μg/g), pentagalloyl glucose (2055 μg/g) and galloyl-valoneic acid dilactone (56 μg/g) and two unknown compounds (55 μg/g). The ellagic acid derivatives are valoneic acid dilactone (250 μg/g), ellagic acid dimer dehydrated (82.9 μg/g), ellagic acid deoxyhexose (37.4 μg/g) and an unknown compound (40.6 μg/g) [15].

Analysis of the effect of toasting indicates that only 9 gallotannins (methyl gallate, 1 digalloyl-HHDP-glucose, 2 trigalloyl glucose, 2 tetragalloyl glucose, pentagalloyl glucose and the two unknown ones) and 4 ellagic derivatives (valoneic acid dilactone, ellagic acid dimer dehydrated and 2 unknown compounds) are detected after light toasting (165 °C for 35 min), with mean concentrations from 28.9 to 1422 μg/g and from not detected to 129 μg/g, respectively [15]. After medium toasting (185 °C for 45 min) of chestnut wood these compounds decrease with 2 gallotannins and 2 ellagic derivatives at concentrations of 210 and 28.5 μg/g and 193 to 69.5 μg/g, respectively, being found [15]. Some of these compounds (mono, di, tri and pentagalloyl glucose) have been detected in chestnut-derived commercial tannin agents [66,67,68]. The total concentration of gallotannins is greater than that of ellagic derivatives in both dry and lightly toasted wood; however, the concentrations of both groups of compounds in the wood after medium toasting are very similar [15].

#### 2.3.2. *Robinia pseudoacacia* L.

Dry acacia wood (24 months in open air) and with light toasting (165 °C for 20 min) present mainly flavonoid compounds [17,64,69,70,71], followed by condensed tannins and LMWP, not presenting hydrolysable tannins. However, this order is not maintained in the wood after medium toasting (165 °C for 35 min) and medium plus toasting (185 °C for 45 min) [17].

In seasoned wood a great variety of flavonoid compounds are identified (18 compounds) with an average concentration ranging from 39 to more than 32265 μg/g, with a total of over 55959 μg/g [17]. The main flavonoids in dry wood are dihydrorobinetin and robinetin [17,64,69]. These flavonoids decrease on toasting, degrading more with higher intensity toasting, except for fisetin and trihydroxymethoxy flavonol, which increase on toasting or butein and tetrahydroxyaurone which increase with gentler toasting though they degrade at higher temperatures [17]. According to Sanz et al. [17] after light and medium toasting (165 °C for 20 min or 35 min, respectively), 18 compounds are found at lower concentrations, presenting a total flavonoid concentration of 42303 and 21444 μg/g for both degrees of toasting. However, more aggressive toasting (185 °C for 45 min) degrades 10 of the compounds completely with a total concentration of 8690 μg/g. Jordao et al. [7] studied 4 flavonoids (robinetin, fustin, robtin and butin) in dry acacia wood after medium toasting (non-specific). The concentration of butin is 440 μg/g [7] and 308 μg/g [17], that of robinetin 14800 μg/g [7] and 7461 μg/g [17], that of fustin 107 μg/g [7] and 1079 μg/g [17] and that of robtin 381 μg/g [7] and 869 μg/g after medium toasting [17]. Dihydrorobinetin compound is degraded the most during toasting, since it is predominant in dry wood with 300 mg/g but cannot be detected after the most intense toasting [17]. In spite of the decrease during toasting, flavonoids are the main chemical compounds representative of toasted wood.

The composition of acacia wood presents condensed tannins not previously described in oak [17,69,71,72]. Seven tannins have been described, 3 identified as leucorobinetinidin, another 3 as dimeric prorobinetinidin and the last as dimeric prorobinetinidin [17]. Toasting degrades the condensed tannin concentration, decreasing proportionally with increased toasting intensity: 3725 μg/g has been recorded in dry wood, 3209 μg/g in lightly toasted wood, 1137 μg/g in medium toasted wood and 73.7 μg/g in medium plus toasted wood; all of them could be found in the previous situation except in the most intensely toasted wood where only prorobinetinidin is detected [17]. These compounds found in acacia wood play a part in the formation of new compounds during wine aging [73], as well as increasing their antioxidant capacity [74]. However, the organoleptic contribution of these compounds is not fully known at present.

#### 2.3.3. *Prunus avium* L. and *Prunus cerasus* L.

The great difference between cherry and oak wood is that oak heartwood does not contain flavonoid compounds. However, *P. avium* heartwood has a great variety of this family of compounds. Nagarajan and Parmar [75] found 11 flavonoids in the heartwood of *P. cerasus* (dihydrotectochrysin, dihydrowogonin, pinocembrin, sakuranetin, naringenin, aromadendrin, taxifolin, kaempferol, quercetin, tectochrysin and chrysin). Vinciguerra et al. [76] identified 5 flavanones (pinocembrin, pinostrobin, dihydrowogonin, naringenin and sakuranetin), 1 dihydroflavonol (aromadendrin-7-methyl ether) and 2 flavones (chrysin and tectochrysin) in *P. avium* heartwood. McNulty et al. [77] identified 6 flavanones (tectochrysin, sakuranetin, dihydrowagonin, naringenin, dihydrokaempferol and catechin) in *P. avium*.

In reference to the effect of toasting on the composition of this wood catechin and naringenin concentrations of 18.51 and 5.54 µg/g, respectively [8], and of 151 and 829 µg/g in medium toasted wood, respectively [7], have been described. Sanz et al. [8] found quercetin in both dry and toasted cherry wood (801 and 324 µg/g, respectively) and quantified 12 procyanidins (condensed tannins) and another 15 flavonoids in the dry wood. The condensed tannins found in this species differ from those recorded in acacia, as they are procyanidin type in cherry and prorobinetin in acacia. Flavonoids found in dry cherry wood are quantified at 36290 µg/g procyanidins and 22768 µg/g other flavonoids. The main procyanidins are flavan-3-ols (β)-catechin (30150 µg/g), β-type procyanidin dimer (1718 µg/g), β-type procyanidin trimer (1122 µg/g) and other flavonoids are naringenin (7514 µg/g), aromadendrin (4535 µg/g), isosakuranetin (3653 µg/g) and taxilofin (3581 µg/g).

Degradation of these compounds is complete for all the procyanidins except catechin and most of the other flavonoids: only 8 of the 15 quantified could be recorded in dry wood: taxifolin, aromadendrin, eriodictyol, naringenin, isosakuranetin, quercetin, kaempferol and apigenin [8]. The concentration of catechin decreases approximately 30 mg/g on toasting, with quantities of 151 µg/g recorded in toasted wood. The total for the other flavonoids after toasting is 1965 µg/g, the predominant one being naringenin with 829 µg/g, followed by quercetin [8]. The order of importance of the compounds in dry acacia wood is procyanidins, followed by the other flavonoids and finally the LMWP, while these are the main compounds found in toasted wood since the procyanidins and other flavonoids degrade with temperature and the LMWP are formed during this process.

#### 2.3.4. *Fraxinus excelsior* L. and *Fraxinus americana* L.

In ash, an important qualitative difference is the presence of secoiridoids, phenylethanoid glycosides, di and oligolignols, which are undetected in oak or the other woods [18]; tannins have also not been detected in *F. excelsior* or *F. americana* [6,18]. The main components in dry wood (24 months in open air) in *F. excelsior* are phenylethanoid glycosides followed by secoiridoids, di and oligolignols and finally LMWP [18]. However, in the subspecies *F. americana* they are the secoiridoids, di and oligolignols, phenylethanoid glycosides and also finally the LMWP [18]. The dry wood of *F. excelsior* is richer in secoiridoids and phenylethanoid glycosides than *F. americana*, with secoiridoid concentrations of 2260 and 1527 µg/g and phenylethanoid glycoside concentrations of 3645 and 470 µg/g, respectively [18]. However, the richest in di and oligolignols and LMWP after drying is *F. americana* [18]. With reference to the secoiridoids in dry wood, 6 are quantified in *F. excelsior* (oleuropein, ligstroside, ligtroside isomer 1, ligtroside isomer 2, ligtroside hexoside and demethyl ligtroside), while only 3 of those are detected (ligstroside, ligtroside isomer 1 and ligtroside isomer 2) in *F. americana* and also oleoside, which has not been detected in *F. excelsior* [18].

Toasting the wood causes degradation of the secoiridoids, this being greater with increased toasting intensity, meaning that oleuropein is only detected in the wood of both subspecies after light toasting (165º for 35 min) and in addition oleoside in *F. Americana*. When toasting is more intense (185 °C for 45 min) no secoiridoids are detected [18]. Therefore, once treated thermally few differences are present in these woods in comparison with traditional oak.

On the other hand, 10 phenylethanoid glycosides (calcelarioside A and B, verbasoside, cistanoside F, verbascoside, isoverbascoside, eukovoside, 2 β-hydroxyverbascosides and β-methoxylverbascoside) are identified in dry ash wood, with small differences between the subspecies, as 3 of them are not detected in *F. americana* (calcelarioside A and B and eukovoside) and 2 in *F. excelsior* (cistanoside F and 1 β-hydroxyverbascoside) [18]. As in the case of the secoiridoids, these compounds degrade on thermal treatment. After medium toasting the only phenylethanoid glycoside which differentiates it from oak is verbascoside. If the toasting intensity is greater this compound may disappear completely as verbascoside goes from 2716 µg/g to 495 µg/g in *F. excelsior* after light toasting and to 26 µg/g after medium toasting, while this decrease is from 217 to 17.7 and to 31.2 µg/g, respectively, in *F. americana* [18].

Finally, 19 di and oligolignols were quantified in dry ash wood, of which only 16 are found in *F. americana* and 10 in *F. excelsior* [18]. After light toasting 6 are quantified in *F. americana* and 8 in *F. excelsior*, while after medium toasting 3 (cycloolivil, olivil and syringaresinol) are found in both subspecies [18]. These compounds also degrade with thermal treatment except for cycloolivil and syringaresinol which, instead of degrading, increase their concentration more as the toasting intensity becomes stronger [18].

Therefore, all these new compounds found in ash involve a small difference when compared with traditional oaks as after medium toasting (the most common in cooperage) 1 phenylethanoid glycoside and 3 di and oligolignols are the distinguishing compounds of this wood vis-à-vis oak.

## 3. Concluding Remarks

The search for new alternatives to using traditional oaks (*Q. petraea*, *Q. robur* and *Q. alba*) includes new *Quercus*, such as *Q. faginea, Q. pyrenaica, Q. frainetto, Q. oocarpa* and *Q. humboldtii* and other new species rather than *Quercus,* like *Castanea sativa* Mill., *Robinia pseudoacacia* L., *Prunus avium* L. and *P. cereaus* L. and *Fraxinus excelsior* L. and *F. americana* L.

The concentrations of the compounds depend on the drying and toasting conditions of the woods, as well as on the origin of the oak as there is a great variability both within the species and within the forest. Ellagitannins are the most abundant compounds in all the oak woods studied. *Q. frainetto* is the oak with the highest concentration in ellagitannins, *Q. pyrenaica, Q. faginea, Q. stellata* and *Q. oocarpa* have similar concentrations to those found in traditional oaks, in general their concentration is between European and American oaks; however, the concentration in *Q. humboldtii* is close to that of *Q. alba* and lower than in the other oaks. On the other hand, in the woods of other genera it has been observed that *Robinia pseudoacacia* and *Fraxinus* do not possess hydrolysable tannins, and in *Prunus* the amount found is insignificant if we compare it to the genus *Quercus*. Chestnut is the only wood that has the same 8 ellagitannins, vescalagin and castalagin being the most important ones as is the case in the *Quercus* species.

As regards LMWP, although their concentration generally increases on toasting, the composition varies much more depending on both the type of *Quercus* and the species. In all the woods studied the acids increase slightly during toasting, being more marked in the case of aldehydes. In general, the main LMWPs present in the wood of new *Quercus* are ellagic acid followed by gallic acid, as in traditional oak. In woods from species of other genera the results are different to those described for *Quercus*. Ash stands out as not presenting ellagic and gallic acids, the main LMWP found in *Quercus* and chestnut wood, because it is the richest in LMWP, with gallic and ellagic acid as the most important compounds. However, gallic acid is generally higher than ellagic, in contrast to what occurs in *Quercus* species.

As regards other compounds (flavonoids or condensed tannins), though the green and seasoned wood of the new species (*Castanea, Robinia, Prunus* and *Fraxinus*) differs from the traditional oak *Quercus* (*petraea*, *robur* and *alba*) genus and others of the same genus (*faginea, pyrenaica, farnetto, oocarpa* and *humboldtii*), these decrease with the toasting process due to the degradation of this type of compounds.

## Figures and Tables

**Table 1:** Summary of extraction and analysis methods of the compounds studied in different woods.

Extraction Methods			
Sample	Extraction Solvent	Conditions	References
Sawdust (1 g)	0.1L MeOH/W (1:1)	room T, 24 h	[3,8,10,11,12,13,14,15,16,17,18]
Sawdust (10 g)	0.3L MeOH/W (1:1)	room T, 24 h	[19,20]
Sawdust (0.5 g)	0.03L MeOH	sonicated, room T, 30 min	[21]
Sawdust (0.1 g)	0.005L W/acetone (3:7)	stirring, room T, 160 min	[22,23]
Sawdust (1 g)	0.1L W/acetone (3:7)	stirring 150 rpm, room T	[24]
Wood chips (0.5 g)	0.01L W/acetone (3:7)	stirring, room T	[25]
Wood chips (2 g)	0.5L model wine 12% (*v*/*v*) pH 3.5)	dark, 5 min stirring daily, 30days	[7]
Wood chips (50 g)	1 L EtOH/W (55:45) and pH 4.2	20 °C, 180 min	[26,27,28,29,30]
Wood chips (2 g)	0.25L model wine, 12% (*v*/*v*); pH 3.2	dark, room T, 35 days	[31]
Wood chips (6 g)	1L hydro alcoholic solutions (55% (*v*/*v*)	dark, 4 weeks	[32]
**Analytical Methods**			
**Extract Preparation**	**Separation Conditions**	**Detection / Calibration Conditions**	**References**
Filtered, liquid-liquid extraction (diethyl ether/ethyl acetate). Evaporated, redissolved MeOH, MeOH /W	Hypersil ODS C18 at 30 °C. Phosphoric Ac. (0.1%), W-MeOH	DAD:255, 280, 325, 340, 360, 525 nm. Spectra 190–650 nm.	[3,8,10,11,12,13,14,15,16,17,18,19,20,33]
		Pure compounds	
Evaporated, re-dissolved W/EtOH (12%).	Eclipse XDB-C18, Reverse-phase C18 LiChrospher at 40 °C. W, FAc-MeOH	DAD-ESI/MS^n^ 280, 320. DAD 325	[21]
		Pure compounds	
Filtered, concentrated, redissolved W	LiChrospher RP-18e LiChrospher 100, Sephadex LH 20. W, FAc-MeOH	DAD 272 and 254. UV spectra 240 to 400 nm	[7,22,23]
		Pure compounds, ellagitannins from Q. robur	
Freeze-dried	Ultrasphere TM. W, FAc-MeOH	DAD 280	[24]
		Expressed in castalagin	
Filtered, liquid-liquid extraction (diethyl ether/ethyl acetate), evaporated, redissolved MeOH	C18 LiChrospher^®^ 100. W, FAc-MeOH	DAD 280	[25,26,28,29,30]
		Ellagic ac. equivalents	
Filtered	Merck Lichrospher RP18 (5 µm) W, FAc-MeOH	UV-Vis and fluorescence 280, 320, 325, 454 nm.	[26,28,29,30]
		Pure compounds	
Filtered	LiChrosphere RP18 W, FAc-MeOH	UV spectra 200–600 nm.	[31]
		Pure compounds	

MeOH: methanol, EtOH: ethanol, FAc: formic cid, Ac: acid, W: water, T: temperature, min: minutes.

**Table 2:** Mean of the concentration expressed as mg/g of the ellagitannins found in green woods of different botanical origin.

Species	Monomers		Pentosylated Monomers		Dimers		Pentosylated Dimers		Total	% Monomers	Ratio
	Castalagin	Vescalagin	Roburin E	Granidinin	Roburin A	Roburin D	Roburin B	Roburin C		**	
**Alternative woods**											
*Q. pyrenaica* (A) ^[11]^	10.63	3.72	5.75	5.39	1.55*	2.09	1.55*	0.4	29.53	49	2.9
*Q. pyrenaica* (B) ^[11]^	11.25	5.48	5.5	5.11	1.48*	2.09	1.48*	0.17	31.08	54	2.1
*Q. pyrenaica* (C) ^[11]^	12.58	5.48	6.51	4.53	1.30*	2.1	1.30*	0.22	32.72	55	2.3
*Q. pyrenaica* (D) ^[11]^	11.49	4.48	5.74	4.11	1.11*	1.83	1.11*	0.32	29.08	55	2.6
*Q. pyrenaica* (E) ^[19,33]^	8.51	6.66	4.78	4.22	1.35	0.51	0.43	1.66	28.12	54	1.3
*Q. faginea* (A) ^[19,33]^	10.01	7.01	5.79	5.43	0.97	1.2	0.47	1.63	32.51	52	1.4
*Q. humboldtti* (A) ^[12]^	0.64	0.32	0.17	0.26	0.06*	0.427	0.06*	0.07	1.94	49	2
**Traditional *Quercus***											
*Q. petraea* Matts. ^[12,19,33]^	9.06–3.89	8.01–1.84	4.44–0.78	5.47–0.75	0.98–0.43	1.71–0.35	0.46–0.31	1.97–0.30	32.10–8.65	53–66	2.1–1.1
*Q robur* L. ^[19,33]^	13.0–9.37	10.6–6.88	9.20–6.13	6.15–2.12	1.91–1.51	0.95–0.69	0.48–0.44	1.72–1.27	44.01–28.41	54–57	1.4–1.2
*Q. alba* L. ^[12]^	1.19	0.7	0.29	0.38	0.13*	0.67	0.13*	0.13	3.48	54	1.7

*Q. pyrenaica* Willd. from: (A) Gata/Peña de Francia; (B) Guadarrama; (C) Cantabrian mountains; (D) Iberian System; (E) Álava. *Q. faginea* Lam. from: (A) Álava. *Q. humboldtti* Bonpl. from: (A) Colombia. The number superscript in the first column is the reference. *A and B were evaluated together; ** % of the monomers in the total ellagitannins.

**Table 3:** Mean of the concentration expressed as mg/g of the ellagitannins found in seasoned woods of different botanical origin.

Seasoning Time	Species	Monomers		Pentosylated Monomers		Dimers		Pentosylated Dimers		Total	% Monomers**	Ratio
		Castalagin	Vescalagin	Roburin E	Granidinin	Roburin A	Roburin D	Roburin B	Roburin C			
**Oven (0%IH)**	**Alternative woods**											
	*Q. pyrenaica* (F) ^[21]^	1.37	0.56	0.49	0.17	0.06	0.08	0.04	0.04	2.81	69	2.4
	*Castanea sativa* (A) ^[21]^	1.73	1.87	1	0.04	0.05	0.03	0.02	0.02	4.74	76	0.9
	*Prunus avium* (A) ^[21]^	0.04	0	0	0	0	0	0	0	0.04	100	-
	**Traditional *Quercus***											
	*Q. petraea* Matts. ^[21]^	0.9	0.39	0.39	0.12	0.05	0.05	0.04	0.04	1.98	65	2.3
	*Q. robur* L. ^[21]^	1.43	0.85	0.77	0.36	0.09	0.18	0.09	0.16	3.93	58	1.7
	*Q. alba* L. ^[21]^	0.41	0.15	0.15	0.07	0.03	0.03	0.02	0.02	0.88	64	2.7
**Oven (12–14% IH)**	**Alternative woods**											
	*Q. humboldtti* (A) ^[13]^	0.54	0.27	0.17	0.22	0.04*	0.32	0.04*	0.05	1.61	50	2
	**Traditional *Quercus***											
	*Q. petraea* Matts. ^[13]^***	9.94–7.46	11.76–6.64	3.29–2.32	1.98–1.88	2.00–1.36*	4.68–3.86	2.00–1.36*	0.30–0.28	33.86–23.89	64–59	1.1–0.8
	*Q. alba* L. ^[13]^***	1.36	0.9	0.3	0.36	0.18*	0.84	0.18*	0.2	4.13	55	1.5
**Natural (NS)**	**Alternative woods**											
	*Castanea sativa* (B) ^[22]^	20.0	43.2	2.0	2.0	4.5	3.3	0.7	0.6	76.3	83	0.5
	**Traditional *Quercus***											
	*Q. petraea* Matts. ^[22]^	19.3	14.1	9.0	7.7	2.0	5.0	2.6	2.3	62.0	54	1.4
**Natural (12 m)**	**Alternative woods**											
	*Q. pyrenaica* (E) ^[33]^	7.48	2.89	3.21	3.52	0.72	0.62	0.63	0.68	19.75	53	2.6
	*Q. faginea* (A) ^[33]^	9.67	6.66	4.64	4.13	0.2	0.48	0.64	0.55	26.97	61	1.5
	**Traditional *Quercus***											
	*Q. petraea* Matts ^[33]^	6.97	1.84	2.51	0.75	0.43	0.35	0.31	0.82	13.98	63	3.8
	*Q. robur* L. ^[33]^	9.37	6.88	6.13	2.12	1.51	0.69	0.48	1.27	28.45	57	1.4
**Natural (24 m)**	**Alternative woods**											
	*Q. frainetto* (A) ^[24]^	14	15.7	8.7	5.2	12.2	5.2	26	21	108	28	0.9
	*Q. stellata* (A) ^[24]^	29.6	16.3	8.5	6.4	1.9	0	2.3	1.9	66.9	69	1.8
	*Q. oocarpa* (A) ^[24]^	23.7	6.5	2.9	6.2	0	0	0	0	39.3	77	3.6
	*Q. pyrenaica* (G) ^[23]^	26.93	30.42	10.67	5.35	ns	4.53	ns	ns	77.9	74	0.9
	*Q. pyrenaica* (G) ^[25]^	5.96	5.86	1.59	1.58	0.51	1.1	0.65	0.62	17.87	66	1
	*Q. pyrenaica* (H) ^[23]^	19.48	10.88	7.49	7.35	ns	9.08	ns	ns	54.28	56	1.8
	*Castanea sativa* (B) ^[24]^	17.4	22.6	0	tr	3.8	0	tr	0	43.8	91	0.8
	*Castanea sativa* (B) ^[15]^	17.37	15.82	2.78	2.37	2.71	2.68	ns	ns	43.73	76	1.1
	*Castanea sativa* (C) ^[25]^	6.71	20.3	2.91	0.34	0.6	0.13	0.33	0.04	31.36	86	0.3
	**Traditional *Quercus***											
	*Q. petraea* Matts. ^[23,24]^	24.81–12.4	21.80–8.70	9.60–4.70	13.50–5.80	2.40–ns	3.81–0.40	2.60–ns	2.1–ns	80.62–32.0	66–58	1.4–1.1
	*Q. robur* L. ^[24]^	30.1	26.7	4.00	8.5	8.7	2.3	3.6	3.5	87.4	65	1.1
	*Q. alba* L. ^[23,24]^	26.40–2.49	6.44–1.89	2.8–nd	tr–nd	tr–ns	0–nd	tr–ns	tr–ns	35.64–4.38	100–87	6.9–0.4
**Natural (36 m)**	**Alternative Woods**											
	*Q. pyrenaica* (E) ^[16]^	4.54	1.68	3.87	3.1	0.73	1.74	0.28	0.16	16.1	39	2.7
	*Q. faginea* (A) ^[16]^	8.18	2.76	5.85	3.21	1.51	2.12	0.31	0.17	24.11	45	3
	**Traditional *Quercus***											
	*Q. petraea* Matts. ^[16]^	12.50–3.43	7.96–1.85	7.98–2.74	4.83–1.81	2.43–0.50	2.58–1.07	0.32–0.15	0.21–nd	38.8–11.6	53–46	1.9–1.6
	*Q. robur* L. ^[16]^	6.68–6.11	5.00–4.62	5.26–3.51	3.69–2.03	1.27–1.09	2.52–1.02	0.26–0.18	0.24–0.14	24.9–18.7	47–57	1.3
	*Q. alba* L. ^[16]^	2.86	1.14	1.75	1.05	0.23	0.84	0.12	nd	7.99	50	2.5

*Q. pyrenaica* Willd. from: (E) Álava; (F) north-west of Spain; (G) Gerês forest of Portugal; (H) Guarda forest of Portugal. *Castanea sativa* Mill. from: (A) Lugo; (B) France; (C) Gerês forest of Portugal. *Prunus avium* from: (A) Lugo. *Q. humboldtti* Bonpl. from: (A) Colombia. *Q. faginea* Lam. from: (A) Álava. *Q. frainetto* Ten. from: (A) Hungary. *Q. stellata* Wangenh. from: (A) Missouri. *Q. oocarpa* Liebm. from: (A) Costa Rica. The number superscript in the second column is the reference. IH: Internal humidity; NS: non specific; ns: not study compound; m: months; tr: traces; nd: not detected; * A and B were evaluated together; ** % of the monomers in the total ellagitannins *** results given by these authors but with 30 months of natural seasoning.

**Table 4:** Mean of the concentration expressed as mg/g of the ellagitannins found in woods of different botanical origin after toasting.

Heat Treat	Species	Seasoning Time	Monomers		Pentosylated Monomers		Dimers		Pentosylated Dimers		Total	% Monomers **	Ratio
			Castalagin	Vescalagin	Roburin E	Granidinin	Roburin A	Roburin D	Roburin B	Roburin C			
160–170 °C 20 min	**Alternative Woods**												
	*Q. pyrenaica* (E) ^[23]^	NS, 24m	15.2	14.62	8.8	4.51	ns	2.53	ns	ns	45.66	65	1
	*Q. pyrenaica* (H) ^[23]^		19.74	11.37	12.37	3.57	ns	nd	ns	ns	47.05	66	1.7
	*Q. pyrenaica* (E) ^[16]^	NS, 36m	2.77	0.37	1.66	0.42	0.41	0.38	0.21	0.15	6.37	49	7.5
	*Q. faginea* (A) ^[16]^		4.68	0.96	2.27	nd	0.24	0.8	0.25	0.14	9.34	60	4.9
	**Traditional *Quercus***												
	*Q. petraea* Matts. ^[23]^	NS, 24m	22.76–20.10	17.61–12.62	10.68–2.42	4.34–2.10	ns	1.37–0.70	ns	ns	56.76–37.94	86–71	1.6–1.3
	*Q. alba* L. ^[23]^		0.44–0.37	5.28–1.23	0.17–nd	nd	ns	nd	ns	ns	5.89–1.60	100–97	0.3–0.1
	*Q. petraea* Matts. ^[16]^	NS, 36m	3.79–1.75	1.02–0.36	2.23–0.75	0.59–0.13	0.50–0.20	0.44–0.16	0.22–0.10	0.17–0.08	8.96–3.53	60–54	4.9–3.7
	*Q. robur* L. ^[16]^		5.44–3.60	1.15–0.89	2.39–1.80	0.66–0.28	0.47–0.41	0.50–0.45	0.20–0.17	0.19–0.12	11.00–7.72	58–60	4.7–4.0
	*Q. alba* L. ^[16]^		nd	nd	nd	nd	nd	nd	nd	nd	nd	nd	
160–170 °C 30 min	**Alternative woods**	NS, 24m											
	*Q. pyrenaica* (G) ^[25]^		1.7	0.62	0.46	0.41	0.32	0.34	0.27	0.2	4.32	54	2.7
	*Castanea sativa* (C) ^[25]^		4.59	4.32	1.09	0.15	0.22	0.06	0.06	0.02	10.51	85	1.1
165 °C 35 min	**Alternative woods**	NS, 24m											
	*Castanea sativa* (B) ^[15]^		6.56	1.03	0.88	0.96	0.33	0.4	ns	ns	10.15	75	6.4
Medium Intensity	**Alternative woods**	NS											
	*Q. pyrenaica* (I) ^[7]^		4.07	3.22	ns	ns	ns	ns	ns	ns	7.29	ns	ns
	*Robinia* p. (A) ^[7]^		nd	nd	ns	ns	ns	ns	ns	ns	ns	ns	ns
	*Prunus avium* (B) ^[7]^		nd	nd									
	**Traditional *Quercus***												
	*Q. petraea* Matts. ^[7]^		3.12	2.4	ns	ns	ns	ns	ns	ns	5.52	ns	ns
	*Q. alba* L. ^[7]^		0.68	0.78	ns	ns	ns	ns	ns	ns	1.46	ns	ns
185 °C 45 min	**Alternative woods**	NS, 24m											
	*Castanea sativa* (B) ^[15]^		0.55	0.11	nd	nd	nd	nd	ns	ns	0.66	100	5
200 °C 140 min	**Alternative woods**	oven 0% IH											
	*Q. humboldtti* (A^)^^[13]^		0.03	0.01	0.01	0.03	0.00*	0.04	0.00*	0.00	0.12	33	3
	**Traditional *Quercus***	NS, 30m											
	*Q. petraea* Matts. ^[13]^		3.34–1.98	1.46–0.87	0.30–0.16	0.31–0.09	0.24–0.14*	0.38–0.18	0.24–0.14*	0.04–0.03	5.83–3.68	82–77	2.3
	*Q. alba* L. ^[58]^		0.35	0.08	0.02	0.04	0.02*	0.04	0.02*	0.01	0.56	77	4.38
250–260 °C 27 min	**Alternative woods**	NS, 24m											
	*Q. pyrenaica* (G) ^[23]^		7.72	4.81	nd	0.85	ns	nd	ns	ns	13.38	94	1.6
	*Q. pyrenaica* (H) ^[23]^		17.69	4.9	4.99	1.75	ns	nd	ns	ns	29.33	77	3.6
	**Traditional *Quercus***												
	*Q. petraea* Matt. ^[23]^		11.60–3.93	12.33–6.52	nd	1.26–1.14	ns	nd	ns	ns	25.19–11.59	90–95	0.9–0.6
	*Q. alba* L. ^[23]^		0.35–0.21	5.07–0.69	nd	nd	ns	nd	ns	ns	5.42–0.90	100	0.5–0.0

*Q. pyrenaica* Willd. from: (E) Álava; (G) Gerês forest of Portugal; (H) Guarda forest of Portugal; (I) Portugal. *Q. faginea* Lam. from: (A) Álava. *Castanea sativa* Mill. from: (B) France; (C) Gerês forest of Portugal. *Robinia pseudoacacia* L. (A). *Prunus avium* from: (B) central France. *Q. humboldtti* Bonpl. from: (A) Colombia. NS: Natural seasoning, m: months, ns: not studied compound; nd: not detected; * A and B were evaluated together ** % of the monomers in the total ellagitannins; castalagin/vescalagin ratio. IH: Internal humidity. The number superscript in the second column is the reference.

**Table 5:** Mean of the concentration expressed as µg/g of the low molecular weight phenolic compounds found in green woods of different botanical origin.

Species	Acids						Aldehydes					Coumarins		Total *
	Ellagic	Gallic	Syringic	Vanillic	Ferulic	Total *	Coniferyl	Sinapic	Syringic	Vanillin	Total *	Scopoletin	Aesculetin	
**Alternative woods**														
*Q. pyrenaica* (A) ^[11,14]^	696	180	6.5	5,0	ns	888	4.07	4.74	8.94	5.87	24	1.08	ns	912
*Q. pyrenaica* (B) ^[11,14]^	626	112	5.01	4.33	ns	747	3.9	4.24	8.52	7.22	24	4.42	ns	776
*Q. pyrenaica* (C) ^[11,14]^	877	143	7.39	3.88	ns	1031	3.84	3.86	10.32	5.63	24	5.61	ns	1061
*Q. pyrenaica* (D) ^[11]^	890	99.5	4.93	3.3	ns	998	2.34	3.2	7.27	3.61	16	4.23	ns	1018
*Q. pyrenaica* (D) ^[14]^	890	124	4.93	3.8	ns	1023	3.06	5.62	7.67	3.69	20	9.55	ns	1052
*Q. pyrenaica* (E) ^[20,33]^	183	63	1.59	1.84	0.51	250	3.26	2.48	1.79	1.91	9	2.04	0.83	262
*Q. pyrenaica* (J) ^[14]^	692	83.5	4.19	2.15	ns	782	3.48	3.86	10.42	4.15	22	6.52	ns	810
*Q. pyrenaica* (K) ^[14]^	654	15.99	3.88	1.57	ns	675	2.99	3.33	6.61	3.36	16	5.39	ns	697
*Q. faginea* (A) ^[20,33]^	213	176	1.66	1.72	0.5	393	4.26	3.53	2.03	1.54	11	1.35	1.26	407
*Q. humboldtti* (A) ^[10]^	286.88	77.6	3.56	4.18	ns	372	2.52	4.9	9.43	6.33	23	ns	ns	395
**Traditional *Quercus***														
*Q. petraea* Matts. ^[10,20,33]^	560.73–195	145–16.17	8.40–2.31	6.66–1.9	1.16–ns	722–215	3.85–0.78	4.92–2.48	12.29–3.55	5.30–2.77	10–26	1.07–ns	2.54–ns	752–225
*Q. robur* L. ^[20,33]^	186–253	100–341	2.69–9.51	1.98–4.74	1.3–1.06	292–609	3.77–6.32	3.94–4.29	3.75–11.4	2.91–6.81	14–29	2.27	1.83	647
*Q. alba* L. ^[10]^	352.72	87.9	6.09	6.18	ns	453	3.08	5.57	14.4	9.27	32	ns	ns	485

*Q. pyrenaica* Willd. from: (A) Gata/Peña de Francia; (B) Guadarrama; (C) Cantabrian mountains; (D) Iberian System; (E) Álava; (J) Alitse–Maragatería; (K) Gredos/Ávila mountains. *Q. faginea* Lam. from: (A) Álava. *Q. humboldtti* Bonpl. from: (A) Colombia. The number superscript in the first column is the reference. *: total is calculated as the sum of all those presented in the table; ns: not studied compound.

**Table 6:** Mean of the concentration expressed as µg/g of the low molecular weight phenolic compounds found in seasoned woods of different botanical origin.

Seasoning (Time)	Species	Acids					Total *	Aldehydes				Total *	Coumarins		Total *
		Ellagic	Gallic.	Syringic	Vanillic	Ferulic		Coniferyl	Sinapic	Syringic	Vanillin		Scopoletin	Aesculetin	
Oven (0% IH)	**Alternative woods**														
	*Q. pyrenaica* (F) ^[21]^	137.35	72.33	36.2	16.13	5.36	267	18.73	39.53	67.67	25.74	152	273.96	ns	693
	*Castanea sativa* (A) ^[21]^	103.59	267.23	76.98	56.74	12.82	517	27.87	92.28	168.52	63.61	352	285.85	ns	1155
	*Prunus avium* (A) ^[21]^	15.8	31.11	43.94	30.54	14.87	136	332.59	78.72	42.01	30.38	484	0	ns	620
	**Traditional *Quercus***														
	*Q. petraea* Matts. ^[21]^	144.87	72.02	201.09	98.49	15.34	532	37.3	282.6	275.64	20.16	616	252.04	ns	1400
	*Q robur* L. ^[21]^	143.11	238	87.09	108.81	9.91	587	28.6	106.16	152.57	71.23	359	260.03	ns	1206
	*Q. alba* L. ^[21]^	177.19	77.14	37.33	46.17	7.84	346	13.61	27.15	45.3	177.01	263	455.14	ns	1064
Oven (12–14% IH)	**Alternative woods**														
	*Q. humboldtti* (A) ^[13]^	562.05	244.05	3.49	4.05	ns	814	2.47	10.18	5.51	ns*	18	ns	ns	832
	**Traditional *Quercus***														
	*Q. petraea* Matts. ^[13]^**	613.19–597.74	139.28–43.12	8.15–6.33	7.95–4.07	ns	769–651	3.74–2.91	6.41–3.42	12.13–8.90	ns*	22–15	ns	ns	791–666
	*Q. alba* L. ^[13]^**	419.94	8.51	6.54	6.59	ns	442	3.99	5.28	18.15	ns*	27.42	ns	ns	469
Natural (Non-specific)	**Alternative woods**														
	*Q. pyrenaica* (I) ^[27]^	1254.8	545.2	82.6	95	230.6	2208	2.6	7.2	21.4	6.2	37	ns	ns	2246
	*Q. pyrenaica* (I) ^[28]^	1806	1318	107	78	201	3510	4	8	17	5	34	2.01	ns	3546
	*Q. pyrenaica* (I) ^[28]^	2071	771	106	125	230	3303	2	8	25	8	43	1.23	ns	3347
	*Q. pyrenaica* (I) ^[28]^	2679	1094	154	121	197	4245	4	11	31	12	58	0.87	ns	4304
	*Q. pyrenaica* ^[28]^	848	494	86	33	269	1730	3	10	23	8	44	25.15	ns	1799
	*Castanea sativa* (D) ^[29]^	1955.2	3263.2	1215.8	348.4	845.8	7628	0	2.8	1.6	5.2	9.6	1.61	ns	7640
	*Castanea sativa* (E) ^[28]^	1105	2540	234	792	408	5079	0	83	50	160	293	1.07	ns	5373
	*Castanea sativa* ^[22]^	1700	1800	ns	ns	ns	3500	ns	ns	ns	ns	ns	ns	ns	3500
	**Traditional *Quercus***						2256								
	*Q. petraea* Matts. ^[22,28]^	2400–1068	1000–846	78–ns	49–ns	215–ns	3400–2256	6–ns	12–ns	22–ns	12–ns	52–ns	3.45–ns	ns	3400–2308
	*Q. robur* L. ^[28]^	1506	484	81	81	235	2387	2	10	15	2	29	5.28	ns	2421
Natural (12 m)	**Alternative woods**														
	*Q. pyrenaica* (E) ^[33]^	299	489	11.7	5.39	0.72	806	3.97	5.11	9.13	5.91	24	6.4	2.65	839
	*Q. faginea* (A) ^[33]^	340	383	6.39	2.65	0.77	733	3.95	8.69	5.75	3.76	22	3.09	2.1	760
	**Traditional *Quercus***						0					0			
	*Q. petraea* Matts. ^[33]^	224	106	6.97	4.32	0.62	342	5.07	3.91	8.15	5.94	23	0.43	2.22	368
	*Q. robur* L. ^[33]^	253	341	9.51	4.74	1.06	609	6.32	4.29	11.4	6.81	29	2.84	5.62	647
Natural (18 m)	**Alternative woods**														
	*Castanea sativa* (F) ^[26]^	870	5500	2490	560	670	10090	110	10	70	10	200	0.25	ns	10290
	*Castanea sativa* (G) ^[26]^	780	9100	3350	560	640	14430	170	20	100	10	300	0.59	ns	14730
Natural (24 m)	**Alternative woods**														
	*Q. pyrenaica* (G) ^[25]^	296.9	117.78	3.06	8.57	5.36	431	12.96	8.41	14.2	7.91	43.48	ns	ns	475
	*Castanea sativa* (B) ^[15]^	588	6166	7.38	7.11	10.4	6779	8.42	11.8	14	20.5	54.72	1.26	ns	6835
	*Castanea sativa* (C) ^[25]^	325.71	590.54	15.99	74.67	6.76	1014	2.57	5.38	78.73	72.06	159	ns	ns	1172
	*Castanea sativa* (D) ^[31]^	208.75	7801.88	9.58	nd	ns	8020	nd	nd	nd	11.25	11.25	ns	ns	8031
	*Robinia* p. (A) ^[17]^	14.2	27.09	nd	nd	ns	41	nd	nd	nd	nd	nd	ns	ns	41
	*Robinia* p. (B) ^[31]^	88.33	291.04	28.54	nd	ns	408	nd	nd	nd	nd	nd	ns	ns	408
	*Prunus avium* ^[8]^	nd	1.22	nd	2.04	ns	3	nd	nd	nd	nd	nd	2.42	ns	6
	*Prunus cereaus* (A) ^[31]^	193.96	nd	21	13	ns	228	nd	nd	nd	nd	nd	ns	ns	228
	*Fraxinus americana* L. ^[18]^	nd	nd	4.11	16.5	4.02	25	10.6	18.6	20.6	23.4	73.2	ns	ns	98
	*Fraxinus excelsior* L. ^[18]^	nd	nd	2.44	6.04	2.89	11	6.01	9.94	13.8	12	41.75	ns	ns	53
	**Traditional *Quercus***														
	*Q. robur* L. ^[31]^	333.75	3767	25.63	40	ns	4166	nd	nd	nd	nd	nd	ns	ns	4166
Natural (36 m)	**Alternative woods**														
	*Q. pyrenaica* (E) ^[3]^	735	445	15	14.2	2.0	1211	7.5	2.9	21.0	15.0	46	ns	1.9	1259.5
	*Q. faginea* (A) ^[3]^	790	582	11.3	9.2	2.4	1395	4.6	1.9	13.1	6.8	26	ns	1.4	1422.7
	**Traditional *Quercus***														
	*Q. petraea* Matts. ^[3]^	328–547	285–72	12.5–9.6	13.9–11.7	2.8–2.8	858–427	11.7–6.4	3.4–2.2	20.1–12.8	13.5–8.6	49–30	ns	3.7–1.2	911–458
	*Q. robur* L. ^[3]^	736–592	243–181	17.9–8.6	14.8–12.0	4.1–2.7	940–869	10.1–6.2	2.7–1.9	16.7–8.1	10.7–5.6	40–26	ns	2.8–1.2	982–898
	*Q. alba* L. ^[3]^	746	103	24.4	13.4	nd	886.8	5.9	4.2	20.7	13.4	44.2	ns	5.0	936

*Q. pyrenaica* Willd. from: (E) Álava; (F) north-west of Spain; (G) Gerês forest of Portugal; (I) Portugal. *Castanea sativa* Mill. from: (A) Lugo; (B) France; (C) Gerês forest of Portugal; (D) north of Portugal; (E) Portugal; (F) Amarante in northwest of Portugal; (G) Carrazeda in northern Portugal. *Prunus avium* from: (A) Lugo. *Prunus cereaus* from: (A) central France (25 months). *Q. humboldtti* Bonpl. from: (A) Colombia. *Quercus*: *Q. alba* L., *Q. stellata*., *Q. lyrata*. and *Q. bicolor*. *Q. faginea* Lam. from: (A) Álava. *Robinia pseudoacacia* L. (A) and with 25 months (B). The number superscript in the second column is the reference. m: months; *: total is calculated as the sum of all those presented in the table; ns: not studied; ns**: not studied with HPLC.

**Table 7:** Mean of the concentration expressed as µg/g of the low molecular weight phenolic compounds found in woods of different botanical origin after toasting.

Heat Treatment	Species	Seasoning Time (Months)	Acids						Aldehydes					Coumarins		
			Ellagic	Gallic	Syringic	Vanillic	Ferulic	Total *	Coniferyl	Sinapic	Syringic	Vanillin	Total *	Scopoletin	Aesculetin	Total *
100 °C 45 min	**Alternative woods**	Nat (NS)														
	*Q. pyrenaica* (I) ^[27]^		1895	514.6	104.2	146.6	195.4	2856	11.4	26.8	20.4	14.2	73	ns	ns	2929
120—155 °C 25 min	**Alternative woods**	Nat (36 m)														
	*Castanea sativa* (D) ^[30]^		3277	2658.6	820.8	198.8	205	7160	212.8	822.8	119.2	109.4	1264	0.475	ns	8425
	**Traditional *Quercus***															
	*Q. robur* L. ^[30]^		1384	163.6	115.8	66	175.6	1905	330.6	1078.2	129.6	32.8	1571	39.16	ns	3480
	*Q. alba* L. ^[30]^		613.6	78	86.2	38.2	206.4	1022	300	1136.6	123.4	30.2	1590	154.01	ns	2628
150 °C 45 min	**Alternative woods**	Nat (NS)														
	*Q. pyrenaica* (I) ^[27]^		2522.4	413.2	91.4	97.6	183.6	3308	143.4	334	39.4	27	544	ns	ns	3852
160—170 °C 20 min	**Alternative woods**	Nat (24 m)														
	*Q. pyrenaica* (G) ^[23]^		3940	ns	ns	ns	ns	3940	ns	ns	ns	ns	ns	ns	ns	3940
	*Q. pyrenaica* (H) ^[23]^		20500	ns	ns	ns	ns	20500	ns	ns	ns	ns	ns	ns	ns	20500
	*Robinia* p. (A) ^[17]^		2.76	43	nd	nd	ns	46	69.5	57	19.8	8.29	155	ns	ns	201
	**Traditional *Quercus***															
	*Q. petraea* ^[23]^		2600—4420	ns	ns	ns	ns	2600—4420	ns	ns	ns	ns	ns	ns	ns	2600—4420
	*Q. alba* ^[23]^ ^v^		3620—1900	ns	ns	ns	ns	3620—1900	ns	ns	ns	ns	ns	ns	ns	3620—1900
160—170 °C 30 min	**Alternative woods**	Nat (24 m)														
	*Q. pyrenaica* (G) ^[25]^		420.27	88.91	2.00	1.82	2.99	515.99	15.72	26.79	33.55	14.89	91	ns	ns	607
	*Castanea sativa* (C) ^[25]^		441.71	488.54	5.74	34.03	4.19	974.21	11.86	78.50	134.47	153.7	379	ns	ns	1353
Medium intensity	**Alternative woods**	NS														
	*Q. pyrenaica* (I) ^[7]^		613.75	180	27.5	16	ns	837	112.5	ns	13.75	ns**	126	ns	ns	964
	*Robinia* p. (A) ^[7]^		6.13	nd	nd	nd	ns	6	nd	ns	nd	ns**	ns	ns	ns	6
	*Prunus avium* (B) ^[7]^		90	nd	nd	nd	ns	90	nd	ns	nd	ns**	ns	ns	ns	90
	**Traditional *Quercus***															
	*Q. petraea* Matts. ^[7]^		436.25	156.25	55	17.5	ns	665	177.5	ns	13.63	ns**	191	ns	ns	856
	*Q. alba* L. ^[7]^		146.25	nd	103.75	41.25	ns	291	168.75	ns	nd	ns**	169	ns	ns	460
160—170 °C 35 min	**Alternative woods**	Nat (36 m)														
	*Q. pyrenaica* (E) ^[3]^		835	361	44.4	24.5	nd	1264.9	386	265	186	72.3	909	ns	1.6	2176
	*Q. faginea* (A) ^[3]^		955	436	37	29.9	nd	1457.9	312	174	119	64.6	670	ns	4.9	2132
	*Castanea sativa* (B) ^[15]^		1406	8211	51.2	28.8	28.9	9726	337	1219	264	163	1983	6.73	ns	11709
	*Robinia* p. (A) ^[17]^		1.01	83.3	51.8	nd	ns	136	276	239	88.3	46	649	ns	ns	785
	*Fraxinus americana* L. ^[18]^		nd	nd	61	71.6	17.2	150	588	672	260	245	1765	ns	ns	1915
	*Fraxinus excelsior* L. ^[18]^		nd	nd	161	66.6	34.7	262	388	773	560	313	2034	ns	ns	2296
	**Traditional *Quercus***															
	*Q. petraea* Matts. ^[3]^		916—729	450—196	48.5—29.5	30.1—22.4	nd	1418—977	545—392	342—281	189—136	91.8—56.8	1168—866	ns	4.2—1.97	2288—2147
	*Q. robur* L. ^[3]^		1176—895	490—234	43.5—31.7	22.8—21.4	nd	1731—1184	424—327	212—252	154—138	67.2—57.9	881—751	ns	1.68—2.1	2483—2067
	*Q. alba* L. ^[3]^		753	96	168	35.5	nd	1053	675	595	536	151	1957	ns	1.45	3011
160 °C 120 min	**Alternative woods**															
	*Castanea sativa* (D) ^[31]^	Nat (22 m)	315.42	3017.50	59.79	37.08	ns	3430	161.67	325.63	228.33	126.46	842	ns	ns	4272
	*Robinia* p. (B) ^[31]^	Nat (25 m)	nd	126.04	nd	nd	ns	126	nd	24.38	nd	nd	24	ns	ns	150
	*Prunus cereaus* (A) ^[31]^		88.96	nd	37.50	26.88	ns	153	41.25	155.63	38.54	56.67	292	ns	ns	445
	**Traditional *Quercus***															
	*Q. robur* L. ^[31]^	Nat (32 m)	742.50	1804.17	33.33	38.13	ns	2618	19.17	50.21	18.54	19.17	107	ns	ns	2725
185 °C 20 min	**Alternative woods**	Nat (NS)														
	*Castanea sativa* (I) ^[32]^		ns	27451.67	193.33	nd	ns	27645	1340.00	1796.67	945.00	656.67	4738	ns	ns	32383
	*Prunus avium* (C) ^[32]^		ns	nd	145.00	nd	ns	145	340.00	593.33	308.33	188.33	1430	ns	ns	1575
	*Fagus sylvatica* (A) ^[32]^		ns	nd	111.67	nd	ns	112	333.33	358.33	168.33	173.33	1033	ns	ns	1145
	*Fraxinus excelior* (A) ^[32]^		ns	nd	75.00	nd	ns	75	663.33	716.67	245.00	221.67	1847	ns	ns	1922
	*Alnus glutinosa* (A) ^[32]^		ns	nd	228.33	276.67	ns	505	361.67	348.33	166.67	138.33	1015	ns	ns	1520
	**Traditional *Quercus***															
	*Q. robur* L. ^[32]^		ns	1793.33	146.67	nd	ns	1940	818.33	1070.00	475.00	211.67	2575	ns	ns	4515
185 °C 45 min	**Alternative woods**	Nat (24 m)														
	*Castanea sativa* (B) ^[15]^		1801	2361	152	77.5	6.05	4398	328	1230	374	158	2090	16.7	ns	6504
	*Robinia* p. (A) ^[17]^		nd	6.92	120	6.52	ns	133	300	1666	326	71.3	2363	ns	ns	2496
	*Prunus avium* ^[8]^		nd	nd	79.9	9.9	ns	90	215	1637	289	41.9	2183	18.8	ns	2292
	*Fraxinus Americana* ^[18]^		nd	nd	122	99.4	28.7	250	826	1196	461	329	2812	ns	ns	3062
	*Fraxinus excelsior* ^[18]^		nd	nd	220	97.3	46.2	364	557	1358	902	404	3221	ns	ns	3585
185 °C 60 min	**Alternative woods**	Nat (NS)														
	*Castanea sativa* (I) ^[32]^		ns	22746.67	726.67	2490.00	ns	25963	1596.67	3828.33	2491.67	1401.67	9318	ns	ns	35282
	*Prunus avium* (C) ^[32]^		ns	nd	258.33	208.33	ns	467	471.67	1336.67	790.00	313.33	2912	ns	ns	3378
	*Fagus sylvatica* (A) ^[32]^		ns	nd	246.67	206.67	ns	453	618.33	781.67	368.33	321.67	2090	ns	ns	2543
	*Fraxinus excelior* (A) ^[32]^		ns	nd	205.00	nd	ns	205	1010.00	985.00	481.67	396.67	2873	ns	ns	3078
	*Alnus glutinosa* (A) ^[32]^		ns	nd	461.67	373.33	ns	835	626.67	743.33	296.67	283.33	1950	ns	ns	2785
	**Traditional *Quercus***															
	*Q. robur* L. ^[32]^		ns	1731.67	435.00	340.00	ns	2507	1238.33	2596.67	1286.67	596.67	5718	ns	ns	8225
200 °C 140 min	**Alternative woods**	Oven (12—14% H)														
	*Q. humboldtti* (A) ^[10]^		552.5	78.27	33.84	21.06	ns	686	485.45	1181.6	110.9	ns**	1778	ns	ns	2464
	**Traditional *Quercus***															
	*Q. petraea* ^[10]^	Nat (30 m)	1012.1—786.1	161.0—157.3	26.11—34.6	13.1—10.8	ns	1206—985	428.41—339.54	1162.0—867.4	78.68—70.9	ns**	1669—1278	ns	ns	2875—2263
	*Q. alba* ^[10]^		641.7	69.87	19.82	13.86	ns	745	513.97	1007.98	86.23	ns**	1608	ns	ns	2353
200 °C 120 min	**Alternative woods**															
	*Castanea sativa* (D) ^[31]^	Nat (22 m)	368.75	2997.29	126.25	77.08	ns	3569	198.33	478.75	396.88	200.63	1275	ns	ns	4844
	*Robinia* p. (B) ^[31]^	Nat (25 m)	nd	200.21	17.00	nd	ns	217	120.21	108.75	nd	8.54	238	ns	ns	455
	*Prunus cereaus* (A) ^[31]^		nd	nd	63.54	38.75	ns	102	77.29	239.17	95.21	64.58	476	ns	ns	579
	**Traditional *Quercus***															
	*Q. robur* L. ^[31]^	Nat (32 m)	680.83	1473.96	78.75	48.75	ns	2282	173.96	259.58	96.25	68.75	599	ns	ns	2881
240 °C 45 min	**Alternative Woods**	Nat (NS)														
	*Q. pyrenaica* (I) ^[27]^		3205	158.2	153.6	104.4	128.2	ns	269.4	862.6	109.8	31.2	1273	ns	ns	5022
240 °C 120 min	**Alternative Woods**															
	*Castanea sativa* (D) ^[31]^	Nat (22 m)	450.45	2469.17	306.04	137.29	ns	ns	215.83	736.46	773.75	305.21	2031	ns	ns	5394
	*Robinia* p. (B) ^[31]^	Nat (25 m)	nd	148.54	28.13	nd	ns	ns	213.13	226.67	nd	14.17	454	ns	ns	631
	*Prunus cereaus* (A) ^[31]^		nd	nd	194.79	70.21	ns	ns	115.00	619.79	460.00	117.71	1313	ns	ns	1578
	**Traditional *Quercus***															
	*Q. robur* L. ^[31]^	Nat (32 m)	752.50	1199.58	171.88	93.96	ns	ns	264.79	559.58	248.75	128.13	1201	ns	ns	3419
250—260 °C 27 min	**Alternative Woods**	Nat (24 m)														
	*Q. pyrenaica* (G) ^[23]^		13750	ns	ns	ns	ns	13750	ns	ns	ns	ns	ns	ns	ns	13750
	*Q. pyrenaica* (H) ^[23]^		19770	ns	ns	ns	ns	19770	ns	ns	ns	ns	ns	ns	ns	19770
	**Traditional *Quercus***															
	*Q. petraea* ^[23]^		4400—4350	ns	ns	ns	ns	4400—4350	ns	ns	ns	ns	ns	ns	ns	4400—4350
	*Q. alba* ^[23]^		2460—2240	ns	ns	ns	ns	2460—2240	ns	ns	ns	ns	ns	ns	ns	2460—2240

*Q. pyrenaica* Willd. from: (E) Álava; (G) Gerês forest of Portugal; (H) Guarda forest of Portugal; (I) Portugal. *Castanea sativa* Mill. from: (B) France; (C) Gerês forest of Portugal; (D) north of Portugal; (I) Asturias. *Robinia pseudoacacia* L. (A) and with 25 months (B). *Prunus avium* from: (B) from central France; (C) Asturias. *Q. faginea* Lam. from: (A) Álava. *Prunus cereaus* from: (A) central France (25 months). *Fagus sylvatica* L. from: (A) Asturias. *Fraxinus excelior* L. from: (A) Asturias. *Alnus glutinosa* L. from: (A) Asturias. *Q. humboldtti* Bonpl. from: (A) Colombia. The number superscript in the second column is the reference. Nat: Natural seasoning; NS: Nonspecific, m: months.
